# The Neuroprotective Effect of Hemin and the Related Mechanism in Sevoflurane Exposed Neonatal Rats

**DOI:** 10.3389/fnins.2019.00537

**Published:** 2019-05-28

**Authors:** Fan Yang, Yangyang Shan, Zhiyin Tang, Xiuying Wu, Congjie Bi, Yongfang Zhang, Yan Gao, Hongtao Liu

**Affiliations:** ^1^Department of Anesthesiology, Shengjing Hospital, China Medical University, Shenyang, China; ^2^Department of Anesthesiology, Dalian Central Hospital, Dalian, China; ^3^Department of Anesthesiology, The First Affiliated Hospital of Hebei North University, Zhangjiakou, China

**Keywords:** hemin, mitochondria, neuroglobin, neurotoxicity, sevoflurane

## Abstract

**Background:**

Many studies have reported that sevoflurane can increase neuronal apoptosis and result in cognitive deficits in rodents. Although neurotoxicity may be associated with mitochondrial dysfunction and oxidative stress, the exact mechanism remains unclear. In order to evaluate potential treatment therapies, we studied the effects of hemin on neurotoxicity of neonatal rat sevoflurane exposure.

**Methods:**

Postnatal day (P) seven rats were assigned randomly to four groups; (1) group C: non-anesthesia, (2) group H: intraperitoneal hemin (50 mg kg^−1^) treatment on days 5 and 6, (3) group S: 3% sevoflurane exposure for 4 h, and (4) group SH: hemin treatment + sevoflurane exposure. The expression of neuroglobin in neonatal hippocampus was determined by western blot and immunohistochemistry. Neuroglobin was localized by immunofluorescence. Western blot for the expression of cleaved caspase-3 and TUNEL were used to detect neonatal hippocampal apoptosis, and cytochrome *c* was used to evaluate mitochondrial function. Drp-1 and Mfn-2 immunoblotting were used to assess mitochondrial dynamics. The Morris water maze test was performed to detect cognitive function in the rats on P30.

**Results:**

Exposure to sevoflurane increased the expression of cleaved caspase-3, cytochrome *c*, and Drp1 in the neonatal hippocampus and resulted in cognitive deficiency but decreased expression of Mfn2. Hemin reduced apoptosis, improved mitochondrial dynamics and ameliorated the cognitive impairment caused by sevoflurane exposure.

**Conclusion:**

Hemin reduced neuronal apoptosis, improved mitochondrial dynamics and protected against cognitive deficits induced by sevoflurane in neonatal rats. This neuroprotective effect may be achieved by increasing the expression of neuroglobin.

## Introduction

Every year thousands of infants and young children receive general anesthesia. Whether general anesthesia has a detrimental effect on children’s neurodevelopment and cognitive function has attracted much attention in recent years and is also controversial (Sun L.S. et al., 2016). Many preclinical studies have demonstrated that anesthetics cause developmental nerve damage, synaptic plasticity, neuronal apoptosis, and affect adult learning and memory function (Satomoto et al., 2009; Shen et al., 2013; Takaenoki et al., 2014). More and more clinical and experimental studies have shown that anesthetics cause cognitive dysfunction and morphological changes in the brain, particularly when they are used on immature and old brains (Vutskits and Xie, 2016). Although several possibilities have been proposed, including oxidative injury and neuronal apoptosis, the neurotoxic mechanisms remain unclear. Many studies have looked for ways to alleviate the neurotoxicity of neonatal rats caused by exposure to anesthetics (Yonamine et al., 2013; Liao et al., 2014; Pellegrini et al., 2014; Ji et al., 2015; Sun Z. et al., 2016; Perez-Zoghbi et al., 2017; Xia et al., 2017; Xu et al., 2017).

Hemin, as a product of heme oxidation, induces heme oxygenase activity, which contains the heme of iron and a chloride ligand (Yuan et al., 2013). It is generally believed that hemin not only has a neuroprotective effect but also induces the expression of neuroglobin (Ngb) (Zhu et al., 2002), which has neuroprotective effects (Zhang et al., 2013). Burmester et al., 2000 discovered Ngb in (Burmester et al., 2000). Ngb is a specific oxygen carrying globulin mainly located in the brain and retina. Studies have demonstrated that Ngb protects neurons, resists ischemic/anoxic damage, and inhibits neuronal apoptosis. Ngb, as an endogenous protective protein has become a hot topic in cerebral ischemia and traumatic brain injury in previous studies (Song et al., 2014; Ragy et al., 2016). However, the effect and mechanism of Ngb on neurotoxicity caused by anesthetics have not been clarified. Furthermore, the effects of hemin on intoxication in neonatal rats exposed to sevoflurane remain unknown. Although studies have shown that sevoflurane can cause changes in mitochondrial dynamics (Boscolo et al., 2013), the effect of hemin on sevoflurane-induced mitochondrial dynamics remains unclear. Therefore, in this study, we investigated the effect of hemin on mitochondrial dynamic mechanism of sevoflurane neurotoxicity and whether hemin as an inducer of Ngb protects against cognitive dysfunction induced by exposing neonatal rats to sevoflurane.

## Materials and Methods

### Ethical Approval

The Animal Care and Ethics Committee of China Medical University approved this study (2017PS001K). The protocol and study were performed in accordance with the recommendations of the Declaration of Helsinki.

### Experimental Animals

Sprague–Dawley rats were purchased from Changsheng Shengwu (China) and obtained from the Experimental and Research Center of Shengjing Hospital. The animals were raised in an environment of light and dark cycle (lights on from 7:00 am to 7:00 pm) with room temperature maintained at 24 ± 1°C. The rats had free access to water and food. A balanced number of rats was drawn from the same litters to reduce the variability caused by different litters. Eighty-four rats were used in this study. Twenty-four rats were decapitated 18 h after anesthesia, and hippocampi were harvested for western blot. Twenty-four rats were decapitated 18 h after anesthesia, and the brain sections were used for immunohistochemistry and the terminal deoxynucleotidyl transferase dUTP nick end labeling (TUNEL) assay. Thirty-six rats were used for the Morris water maze test at P30. Only male rats were included in behavioral testing to avoid potential variability induced by the estrous cycle. Male rats were raised with their mother until 3 weeks of age.

### Sevoflurane Exposure

The rats were anesthetized with 3% sevoflurane (about 1.5 MAC, *n* = 36) plus 40% oxygen/60% nitrogen for 4 h in an anesthesia chamber on P7. The control group received a gas mixture of 40% oxygen and 60% nitrogen at a rate of 2 L/min in a similar chamber. The temperature in the chamber was maintained with hot water. P7 rats were assigned randomly to four groups; (1) group C: non-anesthesia + vehicle saline, (2) hemin administered group (group H, intraperitoneal injection of hemin, 50 mg⋅kg^−1^ at P5 and P6, 0.1 ml); (3) group S: 3% sevoflurane exposure for 4 h in an O_2_/N_2_ mixture of gas (40/60), and (4) group SH: hemin treatment combined with sevoflurane exposure (group SH, 3% sevoflurane for 4 h plus an intraperitoneal injection of hemin, 50 mg⋅kg^−1^ at P5 and P6, 0.1 ml). The chamber was kept away from light. The rats remained in the chamber ventilated with the mixed gas (40% O_2_/60% N_2_) at a rate of 2 L/min until they were fully awakened. After that, the rats were transferred to their original cages.

### Hemin Administration

Hemin was prepared and stored in the dark. The solution and dose of hemin were prepared according to a previous study (Ragy et al., 2016). Hemin powder (300 mg; Sigma, St. Louis, MO, United States; 51280) was first dissolved in 10 ml of 0.1 M NaOH, adjusted to pH 7.4 with 0.1 M HCl, and diluted with saline to the required volume of 30 ml (Ragy et al., 2016).

### Western Blotting

Rats were decapitated under pentobarbital anesthesia (100 mg/kg i.p.) 18 h after sevoflurane exposure (P8), and hippocampi were harvested on ice for western blot in RIPA lysis buffer (Beyotime, Beijing, China) and protease inhibitor cocktail (Beyotime) The homogenate was centrifuged at 14,000 × *g* at 4°C for 30 min, and the supernatant was removed and stored at −80°C. Protein concentrations were determined with a BCA protein assay kit (Beyotime).

Homogenates of hippocampal protein were mixed with 1 × sodium dodecyl sulfate-polyacrylamide gel electrophoresis (SDS-PAGE) sample loading buffer (Beyotime) and denatured for 3 min at 99°C. A 40 μg aliquot of protein was analyzed by 12% SDS-PAGE and transferred to a polyvinylidene fluoride (PVDF) membrane (Millipore, Bedford, MA, United States). Rabbit anti-Ngb antibody (Proteintech, Wuhan, China; 1:1,000) was used to detect the concentrations of Ngb. Rabbit anti-cleaved caspase-3 (Cell Signaling Technology, Danvers, MA, United States; 1:1,000) was used to detect apoptosis. Rabbit anti-drp1 (Wanleibio, Shanghai, China) and Mfn2 antibody (Proteintech; 1:1,000) were used to evaluate mitochondrial dynamics. The membranes were blocked in TBS containing 0.1% Tween-20 (TBST) and 5% non-fat dry milk for 90 min, and then the membrane was incubated overnight at 4°C with primary antibody in TBST. The membrane was incubated with secondary antibody (Sigma; 1:10,000) for 2 h at room temperature after washing with Tris-Buffered Saline and Tween-20 (TBST). A β-actin antibody (Sigma; 1:5000) was used as the loading control. The protein bands were photographed by a GE chemiluminescence detection system (Imager 600; GE America, Milwaukee, WI, United States), and band densities were quantified with Image J 6.0 (National Institutes of Health, Bethesda, MD, United States). The ratio of target protein to β-actin was recorded and analyzed.

### TUNEL

Rats were anesthetized with pentobarbital sodium and perfused with 4% paraformaldehyde (PFA) 18 h after sevoflurane exposure (P8). The whole brain was removed from the skull and immersed in phosphate-buffered PFA at 4°C for 24 h after decapitation. The slices were treated with 10% fetal bovine serum (FBS) to reduce background staining at room temperature for 30 min. Apoptotic cells were detected by terminal deoxynucleotidyl transferase (TdT) and dUTP (11684795910; Roche, Basel, Switzerland) at 4°C overnight. The coronal sections were cut to a thickness of 2.5 μm. The next morning, the nuclei were stained with DAPI for 5 min at room temperature. TUNEL-positive cells were counted in three fields selected randomly at 400× magnification and photographed using a Nikon C1 microscope (Tokyo, Japan).

### Immunohistochemistry

The entire brain was removed from the fetus, immersed in 4% PFA for 48 h at 4°C, dehydrated in a graded ethanol series, and embedded in paraffin. The sections (2.5 μm) were deparaffinized and then heated in citrate buffer for 7 min. The slices were treated with 10% FBS and 3% hydrogen peroxide to reduce background staining at room temperature. The sections were incubated with rabbit anti-Ngb antibody (1:200, Sigma) in PBS at 4°C overnight in a chamber for detecting Ngb. The next day, the sections were incubated with peroxidase-conjugated secondary antibody for 2 h and washed with PBS for 5 min. Then, the sections were reacted with 3,3′-diaminobenzidine (DAB) staining according to the manufacturer’s instructions. The sections were mounted on slides, counterstained with hematoxylin solution (Solarbio, Beijing, China), and photographed with a Nikon C1 microscope. We randomly selected at least three fields of view for each sample and used NIS-Elements AR Analysis 4.50.00 software to quantify the integrated optical density value of Ngb in the neonatal rat hippocampus. Six rats were in each group for the immunohistochemistry experiment.

### Immunofluorescence Double Staining

The sections (2.5 μm) were deparaffinized and then heated in citrate buffer for 7 min. The slices were treated with 10% FBS and 3% hydrogen peroxide to reduce background staining at room temperature. The slices were incubated with two kinds of primary antibodies (NeuN/Ngb and GFAP/Ngb) simultaneously at 4°C overnight in an immunofluorescence double staining chamber. The slices were incubated with secondary antibodies the next day for 2 h at room temperature. The antibodies included: NeuN antibody (1:200, ab104224, Abcam, Cambridge, MA, United States), GFAP antibody (1:200, 3670s; Cell Signaling Technology, Danvers, MA, United States), and Ngb antibody (1:150, N7162, Sigma). The nuclei were stained with DAPI. We used a Nikon C1 microscope to photograph the location of Ngb.

### Morris Water Maze

On day 30 after birth, the Morris water maze experiment was conducted to evaluate spatial learning and memory ability of the rats. In brief, the water maze (160 cm in diameter and 60 cm depth) consisted of a circular pool and an automatic video and analysis system. The water level on the wall was marked with four water inlet points: east, south, west, and north. The pool was divided into four quadrants, and an escape platform was located 1.5 cm below the surface of water in the center of one of the quadrants. The pool was filled with warm (20°C) opaque water. The pool wall, the bottom of the pool, and the platform were painted black to conceal the platform. The surrounding environment and the reference material on the wall of the water labyrinth remain unchanged.

Rats were tested in the Morris water maze for 6 consecutive days. The positioning navigation test was carried out on days 1–5 and was used to assess spatial learning and memory ability of the rat. The probe trial sessions were conducted four times daily from 9:00 to 12:00 for 5 days. Rats (*n* = 9/group) were placed in the water facing the pool wall to search for the hidden platform in the four quadrants. Escape latency (time used to find the platform) was recorded by a video tracking system. The time limit was 90 s. The rats remained on the platform for 10 s. If the rat did not find the platform within 90 s, escape latency was recorded as 90 s. Then the rats were guided to stand on the concealed platform for 10 s. The spatial probe test was conducted on day 6 to test the ability of the rats to maintain their memory of the platform space after learning to find the platform. The platform was removed after the positioning navigation experiment was completed. After the platform was taken away from the maze, the rats were put into the quadrant opposite the platform and permitted to swim freely for 90 s. The time in the target quadrant was noted. This test reflected the ability of the rats to maintain spatial position memory.

### Statistical Analysis

The statistical analysis was conducted using Graph Pad Prism 7.0 software (Graph Pad Software Inc., San Diego, CA, United States), and all data are presented as mean ± standard deviation. Bartlett’s test was used to test for equal variances and normality of the data was checked with the Shapiro–Wilk test. One-way analysis of variance (ANOVA) followed by the Student–Newman–Keuls *post hoc* test were used to compare the means of each group. The escape latency data and time in the target quadrant on the Morris water maze test were compared using two-way ANOVA followed by the Bonferroni *post hoc* test. *P*-values < 0.05 were considered significant.

## Results

### Sevoflurane Increases Apoptosis in the Hippocampus and Disrupts the Mitochondrial Dynamic Balance

**FIGURE 1:**
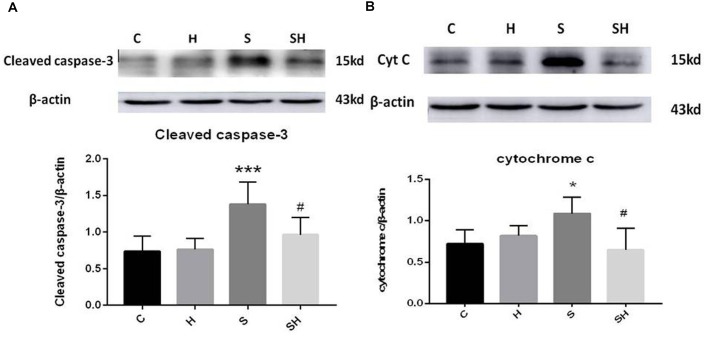
Hemin inhibits the increase of apoptosis caused by sevoflurane Expression and representative image of cleaved caspase-3 **(A)** and cytochrome *c* **(B)** in the neonatal hippocampus 18 h after sevoflurane exposure. (^∗^*P*< 0.05 vs. group C, ^∗∗∗^*P*< 0.001 vs. group C, ^#^*P*< 0.05 vs. group S, *n* = 6/group).

**FIGURE 2:**
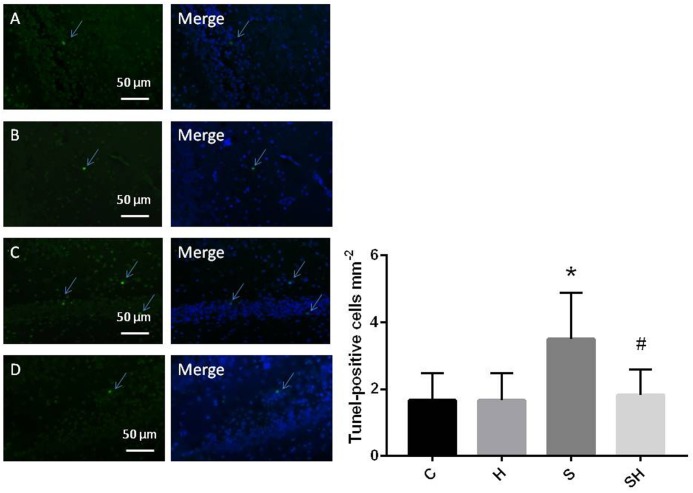
Quantification and detection of TUNEL-positive cells in the hippocampal CA1 region of the neonatal rat brain 18 h after anesthesia Coronal slices for representative TUNEL staining in control **(A)**, H **(B)**, S **(C)**, and SH groups **(D)**. (×400 magnification) (^∗^*P*< 0.05 vs. group C, ^#^*P*< 0.05 vs. group S, *n* = 6/group). Scale bar = 50 μm.

Western blotting was used to detect the expression of cleaved caspase-3 and cytochrome *c* in the hippocampus of rats to evaluate the effect of sevoflurane on apoptosis in the hippocampus of neonatal rats exposed to 3% sevoflurane for 4 h. Compared with the control group, expression of cleaved caspase-3 in group H, group S, and group SH increased by 3.5, 88, and 31%, respectively. Compared with the control group, expression of cytochrome C in group H and group S increased by 13 and 51%, while decreased in group SH by 10%. Exposing the neonatal rats to 3% sevoflurane for 4 h increased apoptosis in the hippocampus. The expression of cleaved caspase-3 and cytochrome *c* in group S increased significantly compared with the control group (*P* < 0.01 and *P* < 0.05) (Figure 1). Compared with the control group, TUNEL-positive cells in group H, group S and group SH increased by 0, 110, and 10%, respectively. The number of apoptotic cells by TUNEL staining increased significantly (*P* < 0.05) compared with the control group in the hippocampal CA1 region of neonatal rats brain 18 h after anesthesia (Figure 2).

**FIGURE 3:**
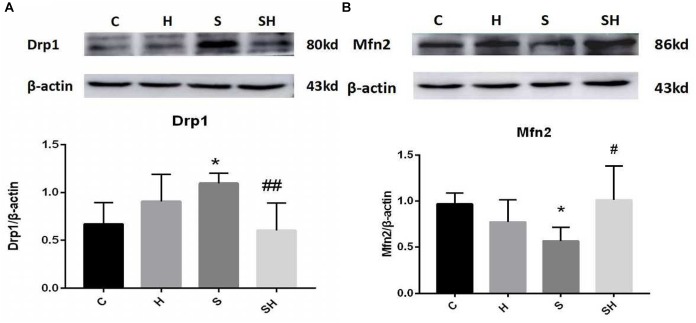
Hemin mitigates the mitochondrial dynamic dysfunction induced by sevoflurane Expression and representative image of Drp1 **(A)** and Mfn2 **(B)** in the neonatal hippocampus 18 h after sevoflurane exposure. (^∗^*P*< 0.05 vs. group C, ^#^*P*< 0.05, ^##^*P*< 0.01 vs. group S, *n* = 6/group).

We examined the expression of Drp1 and Mfn2 by western blotting to investigate mitochondrial dynamics in the neonatal hippocampus. Compared with the control group, expression of Drp1 in group H, group S, and group SH increased by 35, 63, and 10%, respectively. Compared with the control group, expression of Mfn2 in group H and group S decreased by 22 and 36%, while increased by 5% in group SH Figure 3. Sevoflurane anesthesia significantly increased the expression of Drp1 but decreased the expression of Mfn2 (*P* < 0.05), indicating that sevoflurane disrupts the mitochondrial dynamic balance.

### Hemin Increases the Expression of Ngb and Alleviates Apoptosis in the Hippocampus Induced by Sevoflurane Exposure in Neonatal Rats

**FIGURE 4:**
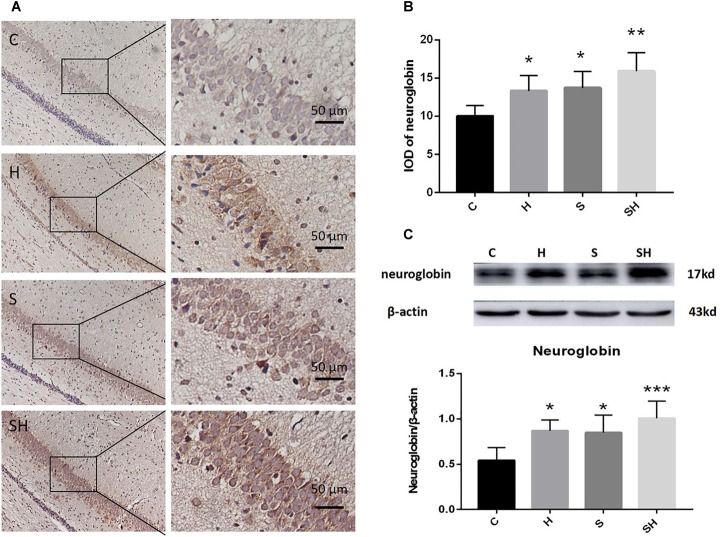
Hemin increases the expression of Ngb. **(A)** Immunohistochemistry of neuroglobin in the neonatal hippocampal CA1 region in each group. Scale bar = 50 μm. **(B)** Integrated optical density of neuroglobin in the neonatal rat hippocampus. (^∗^*P*< 0.05 vs. group C, ^∗∗^*P*< 0.01 vs. group C, *n* = 6/group). **(C)** Expression and representative image of neuroglobin in the neonatal hippocampus 18 h after sevoflurane exposure. (^∗^*P*< 0.05 vs. group C, ^∗∗∗^*P*< 0.001 vs. group C, *n* = 6/group).

Hemin is an inducer of Ngb; thus, Ngb expression was investigated. The immunohistochemistry results in Figure 4A show that the integrated optical densities of Ngb increased significantly in the neonatal hippocampal CA1 region after hemin administration. Compared with the control group, integrated optical density of neuroglobin in group H, group S and group SH increased by 33, 37, and 59%, respectively Figure 4B. Moreover, the western blotting results also demonstrated that the expression of Ngb in groups H and S increased significantly (*P* < 0.05) (Figure 4C). Ngb expression increased more significantly (*P* < 0.01) in the SH group (Figure 4C). Compared with the control group, expression of neuroglobin in group H, group S, and group SH increased by 60, 56, and 86%, respectively. These results suggest that Ngb might be involved in the neurotoxicity of sevoflurane in neonatal rats.

To further show that hemin protects against sevoflurane-induced neurotoxicity in the neonatal rats, we determined the expression of caspase-3 and cytochrome *c* in the hippocampus of neonatal rats exposed to sevoflurane and administered hemin (group SH). The expression levels of cleaved caspase-3 and cytochrome *c* in group SH were not different from those in group C (*P* > 0.05). The expression levels of cleaved caspase-3 and cytochrome *c* in group SH decreased significantly (*P* < 0.05) compared with those in group S (Figure 1). Moreover, the number of apoptotic cells decreased significantly in group SH (*P* < 0.05) compared with group S. The number of apoptotic cells in group SH was not different from that in group C (*P* > 0.05) (Figure 2). These results indicates that hemin induced expression of Ngb and alleviated the neurological damage induced by sevoflurane exposure in neonatal rats.

### Hemin Mitigates the Mitochondrial Dynamic Dysfunction Induced by Sevoflurane

We verified whether hemin is involved in mediating mitochondrial dynamic function by regulating Drp1 and Mfn2 in the neonatal hippocampus 18 h after sevoflurane exposure by western blot. The Mfn2 level was upregulated in the SH group and the Drp1 level was downregulated significantly compared with the S group (Figure 3). These results support that hemin reversed the changes in Drp1 and Mfn2 expression induced by sevoflurane exposure (*P*< 0.05; *P*< 0.01), demonstrating that the dysfunction in mitochondrial dynamics caused by sevoflurane can be mitigated by hemin (Figure 3).

### Localization of Neuroglobin

**FIGURE 5:**
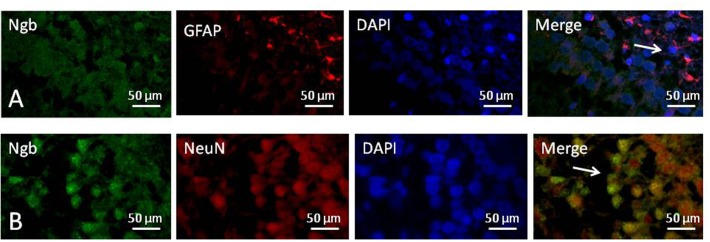
Location of neuroglobin Detection of neuroglobin (Ngb) by immunofluorescence double staining in the hippocampus of the neonatal rat brain 18 h after anesthesia in group H. GFAP (red) co-localized with Ngb (green) shown in **(A)** and NeuN (red) co-localized with Ngb (green) shown in **(B)**. Scale bar = 50 μm.

We used immunofluorescence double staining to study the location of Ngb in the hippocampus of the neonatal rat brain 18 h after anesthesia in group H. The results demonstrated that Ngb was mainly localized in neurons, and a smaller portion was localized in astrocytes (Figure 5).

### Hemin Reduces Sevoflurane-Induced Cognitive Deficiency

**FIGURE 6:**
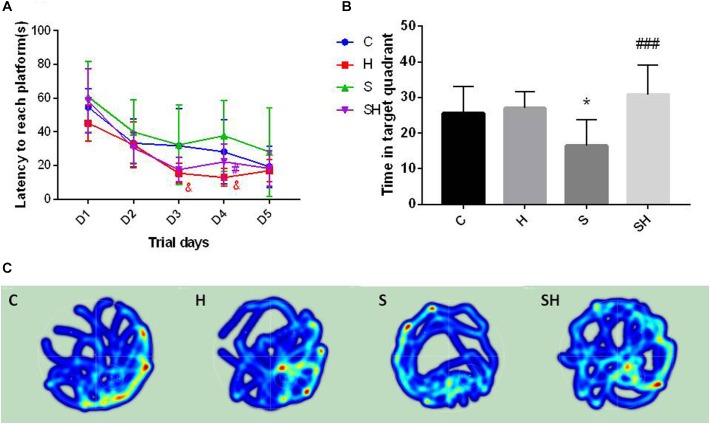
Hemin reduces sevoflurane-induced cognitive deficiency The Morris water maze test was used to assess spatial learning and memory ability of 30 day old rats. **(A)** Escape latency between groups. (&*P*< 0.05 group H vs. group C, ^#^*P*< 0.05 vs. group S, *n* = 9/group) **(B)** Time of target quadrant (^∗^*P*< 0.05 vs. group C, ^###^*P*< 0.001 vs. group S, *n* = 9/group). **(C)** Heatmap of time in the target quadrant.

The Morris water maze was conducted on day 30 after birth to evaluate the effect of hemin on the cognitive impairment caused by a 4 h exposure to sevoflurane in neonatal rats. No difference in motor functional impairment was detected between the groups. Escape latency in orientation of the Morris water maze tended to be no different in groups C and S (*P*> 0.05). However, the time to find the platform in group H on days 3 and 4 of the orientation navigation experiment was shorter than that in group C (*P* < 0.05). Compared with the S group, the time to find the platform on day 4 decreased in the SH group compared with the S group (*P* < 0.05) (Figure 6A). In addition, a significant difference in time spent in the target quadrant was observed between groups C and S (*P* < 0.05). Compared with the control group, time in target quadrant in group H and SH increased by 6 and 21%, respectively, while decreased in group S by 36%. Rats in group S spent less time in the target quadrant than rats in group C. Rats in the group SH spent more time in the target quadrant than those in group S (*P* < 0.001) (Figure 6B). These results indicate that exposing neonatal rats to sevoflurane for 4 h could cause cognitive deficits, and that hemin alleviated the sevoflurane-induced cognitive function impairment in the rats.

The heatmap and swimming track results indicate that rats in group S spent less time in the platform located quadrant compared with the other groups (*P* < 0.05) (Figure 6C), which suggested that the rats were disorientated and had differences in spatial memory. However, there was no significant difference between groups SH and C (*P* > 0.05).

## Discussion

In our study, we evaluated whether hemin alleviated sevoflurane-induced neuronal damage and observed changes in Ngb, cleaved caspase-3, cytochrome *c*, Drp1, and Mfn2. Our results indicate that sevoflurane enhanced the expression levels of cleaved caspase-3 and cytochrome *c* in the hippocampus and caused changes in mitochondrial dynamics by increasing Drp1 and decreasing Mfn2, consistent with previous studies (Boscolo et al., 2013; Twaroski et al., 2015). Hemin increased the expression of Ngb in the brain, alleviated the increase of cleaved caspase-3 and cytochrome *c* caused by sevoflurane, reduced apoptosis in the hippocampus of neonatal rats, and improved the dysfunction in mitochondrial dynamics. These results suggest that sevoflurane damages hippocampal cells by causing mitochondrial apoptosis and mitochondrial dynamic dysfunction. Hemin attenuated the mitochondrial damage and apoptosis induced by sevoflurane by increasing the expression of Ngb, which also improved mitochondrial dynamics. Although there was no significant interaction between the groups (group C vs. group S) in escape latency, the decreased amount of time spent in the target quadrant by group S indicated that sevoflurane exposure for 4 h could cause cognitive deficits in young rats. Hemin alleviated sevoflurane-induced cognitive impairment in young rats.

We used 3% sevoflurane by inhalation because 3% sevoflurane exposure does not induce hypoxia or respiratory depression (Satomoto et al., 2009; Yonamine et al., 2013). Previous studies have shown that a repeated 2-h sevoflurane exposure repeated for 3 days or a single 6-h exposure can cause cognitive deficits in young rats (Satomoto et al., 2009; Murphy and Baxter, 2013; Pellegrini et al., 2014; Amrock et al., 2015; Ji et al., 2015; Sun Z. et al., 2016; Makaryus et al., 2018); we found that a single 4-h inhalation of 3% sevoflurane also caused cognitive impairment.

Previous studies have indicated that anesthetics cause mitochondrial dysfunction, impair the balance of mitochondrial dynamics, disrupt the integrity of mitochondrial structures, and increase ROS (Kodama et al., 2011; Sanchez et al., 2011; Boscolo et al., 2013; Amrock et al., 2015; Vutskits and Xie, 2016; Lu et al., 2017; McCann and de Graaff, 2017). Many studies have investigated ways to reduce the neurotoxicity of sevoflurane, such as the NADPH oxidase inhibitor apocynin, coenzyme Q10, dexmedetomidine, tanshinone IIA, erythropoietin, curcumin, and hydrogen gas, etc., which mitigate anesthesia-induced neurotoxicity and cognitive impairment (Yonamine et al., 2013; Liao et al., 2014; Pellegrini et al., 2014; Ji et al., 2015; Sun Z. et al., 2016; Perez-Zoghbi et al., 2017; Xia et al., 2017; Xu et al., 2017). Some studies have indicated that hemin induces Ngb expression, which protects the brain against focal cerebral hypoxia and ischemia (Wang et al., 2008; Song et al., 2014). However, no direct evidence has indicated the effect of hemin on intoxication by sevoflurane. The underlying mechanism of this neuroprotective effect needs to be elucidated.

Hemin protects mitochondrial function by inhibiting mitochondrial fission and apoptosis, preventing oxidative stress damage, and improving cognitive impairment (Wang et al., 2017; Han et al., 2018; Ye et al., 2018). A study reported that administering a low dose of hemin inhibits endogenous cell apoptosis and has a neuroprotective effect in OGD-treated SH-SY5Y cells (Wang et al., 2017). Another study demonstrated that pharmacological modulation of the heme oxygenase-1 pathway offers protection against intermittent hypoxia-induced cardiac dysfunction and myocardial fibrosis by inhibiting cell apoptosis and mitochondrial fission (Han et al., 2018). Hemin also attenuats cell death and oxidative stress induced by Pb acetate in the developing rat brain (Ye et al., 2018). Consistent with previous research, our study suggests that hemin improved mitochondrial damage and apoptosis caused by sevoflurane exposure and improved the dysfunction in mitochondrial dynamics. This protective effect may be related to higher Ngb levels. According to the previous studies we choose a dose of 50 mg/kg at P5 and P6 intraperitoneally before exposed to sevoflurane. A study suggested that hemin was injected at a dose of 50 mg/kg body weight every other day by intraperitoneal injection for 3 weeks (50 mg/mL dissolved in phosphate-buffered saline, pH 7.4) (Worou et al., 2011). Another study recommended hemin at a dose level of 50 mg/kg body weight intraperitoneally 12 h before being exposed to water immersion restraint stress (Ragy et al., 2016). So according to previous we choose a dose of 50 mg/kg at P5 and P6 intraperitoneally before exposed to sevoflurane.

Neuroglobin is a specific oxygen-binding globin protein first discovered in 2000 that has been verified to play an endogenous neuroprotective molecule against ischemia, hypoxia, oxidative stress damage, and related neurological disorders (Burmester et al., 2000; Qiu and Chen, 2014; Nair et al., 2018; Van Acker et al., 2018). Several studies have demonstrated that Ngb reduces ROS generation, preserves mitochondrial ATP production, resets the level of mitochondrial cytochrome *c*, participates in mitochondrial-mediated cell death signaling, and acts as a regulator of signal transduction in the brain (Wakasugi et al., 2005; Yu et al., 2009; Antao et al., 2010; Yu et al., 2016). Moreover, other studies have reported that Ngb decreases mitochondrial damage, protects mitochondrial function, and improves neuronal apoptosis (Li et al., 2013; Gorgun et al., 2014; Lan et al., 2014). However, the detailed mechanisms of the neuroprotective effects between Ngb and mitochondrial proteins against the neurotoxicity of sevoflurane exposure remain to be further elucidated (Yu et al., 2013). Some studies have found that Ngb is localized in neurons, while other studies have suggested that Ngb is also expressed in astrocytes. We performed immunofluorescence double staining and found that Ngb is mainly localized in neurons. The antioxidant and antiapoptotic molecular properties of Ngb may be used as a potential treatment for alleviating neuronal damage, and its increased expression after brain injury may have endogenous neuroprotective effects. Perhaps in the future, we could improve the expression of Ngb by pharmacological induction or gene therapy to alleviate the brain damage caused by sevoflurane exposure (Baez et al., 2016; Guidolin et al., 2016). Interestingly, the expression level of Ngb in the sevoflurane group was also elevated, which may indicate an endogenous protective effect of Ngb. In addition, we were surprised that the hemin group had significantly improved cognitive function compared with the control group, which may be related to axon regeneration promoted by Ngb. Xiong et al. (2018) reported that Ngb promotes neurite regeneration, which is essential for functional recovery of injured neurons in various neurological diseases. This may be one of the reasons that hemin alleviated sevoflurane-induced cognitive impairment in young rats.

Mitochondria are remarkably dynamic organelles that are remodeled by division and fusion. The balance between these two opposite processes plays an important role in mitochondrial morphology and functional stability. The protein associated with division is Dpr-1, which promotes mitochondrial division. The fusion-related proteins are Mfn1 and Mfn2 and OPA1, which regulate mitochondrial fusion. Studies have shown that anesthetics cause mitochondrial dysfunction, impair the balance of mitochondrial dynamics, disrupt the integrity of mitochondrial structures, and increase ROS (Kodama et al., 2011; Sanchez et al., 2011; Boscolo et al., 2013; Amrock et al., 2015; Vutskits and Xie, 2016; Lu et al., 2017; McCann and de Graaff, 2017). These are important factors in the neurotoxicity of general anesthetics during brain development. Therefore, protecting the integrity and structure of the mitochondria, scavenging oxygen free radicals, and preventing mitochondrial dysfunction are potential therapeutic strategies to mitigate the neurotoxicity of anesthetics. Our study is limited, as we assessed the concentrations of cleaved caspase-3, cytochrome *c*, Drp1, and Mfn2 in the hippocampus, but not in the cortex, of young rats because the hippocampus plays crucial roles in learning and memory and is vulnerable to external damage. Another limitation of our study is only to observe the effect of hemin administration before sevoflurane exposure, but not after sevoflurane exposure. These need to be further explored in future experiments.

## Conclusion

In conclusion, we demonstrated that the Ngb inducer hemin reduced apoptosis in the hippocampus, improved mitochondrial dysfunction, and protected against cognitive impairment in neonatal rats exposed to sevoflurane. This neuroprotective effect may be achieved by increasing the expression of Ngb. In the future, we may improve the expression of Ngb to alleviate the neurotoxicity of anesthetics by pharmacological induction or gene therapy.

## Ethics Statement

The Animal Care and Ethics Committee of China Medical University approved this study (2017PS001K). The protocol and study were performed in accordance with the recommendations of the Declaration of Helsinki.

## Author Contributions

FY and HL designed and planned the study. All authors conducted the study, analyzed the data, and revised the manuscript. FY wrote the manuscript.

## Conflict of Interest Statement

The authors declare that the research was conducted in the absence of any commercial or financial relationships that could be construed as a potential conflict of interest.
